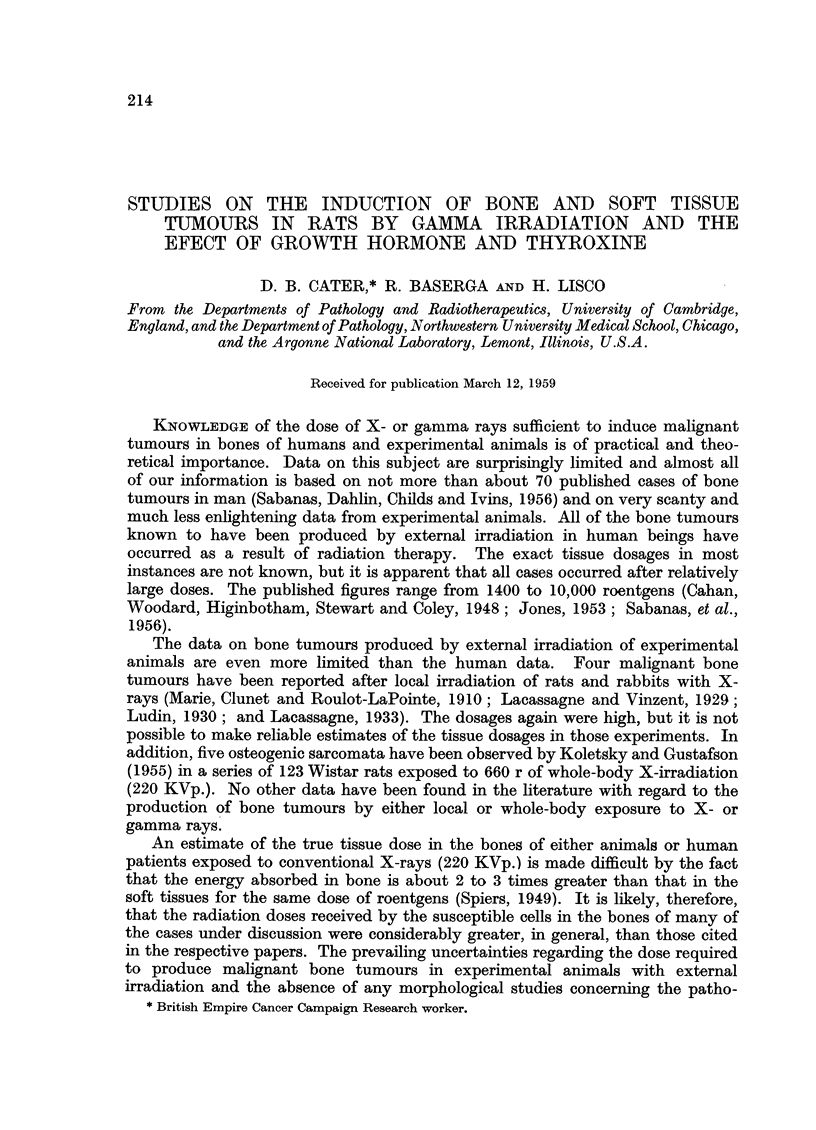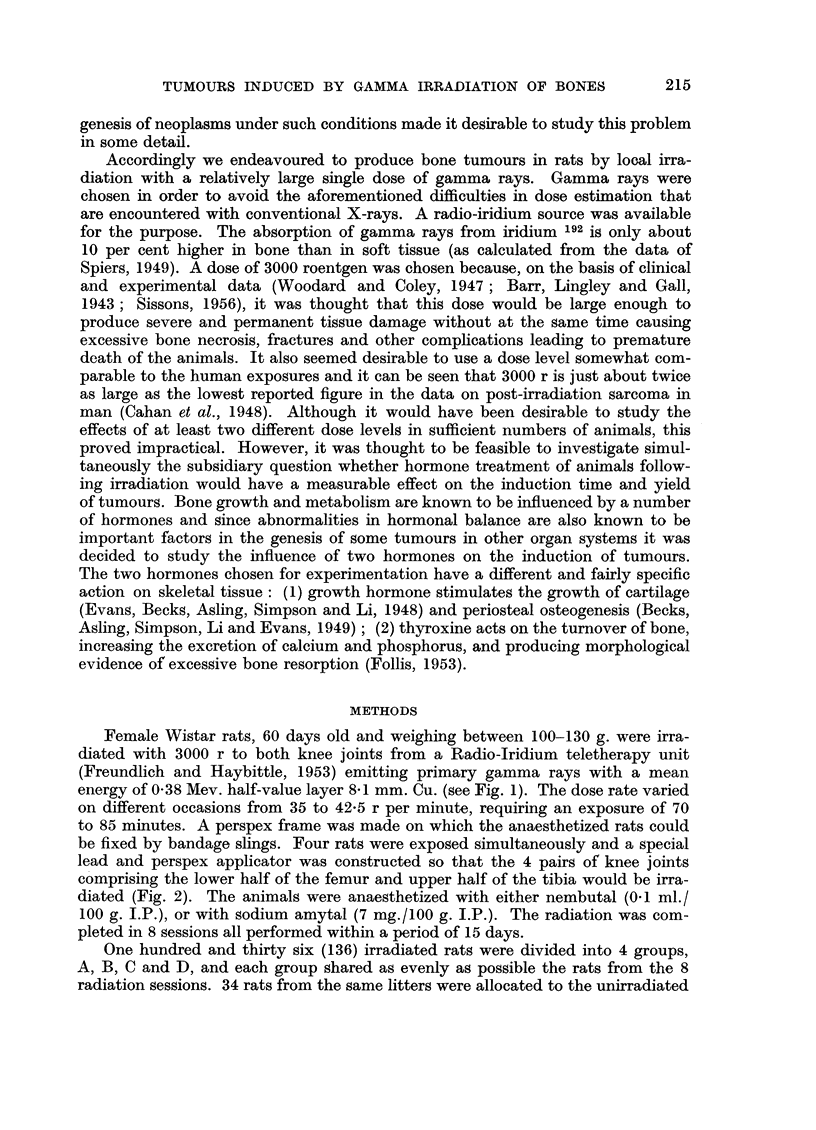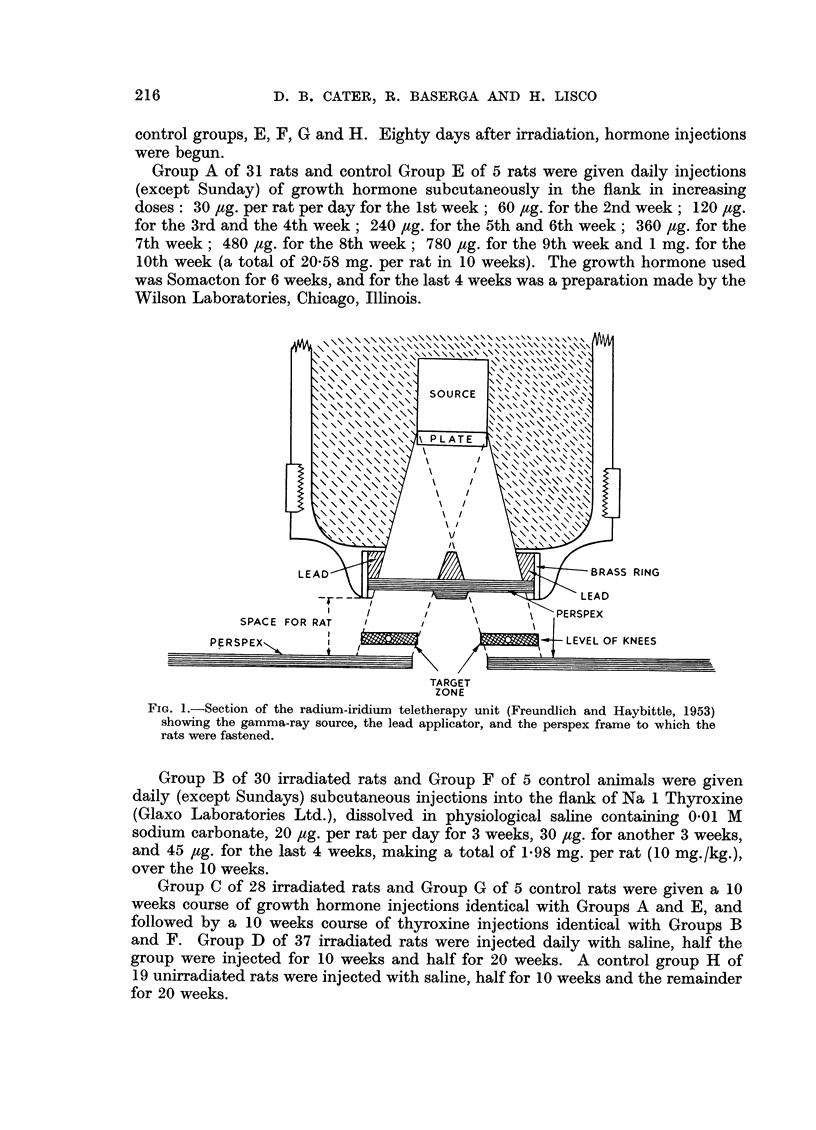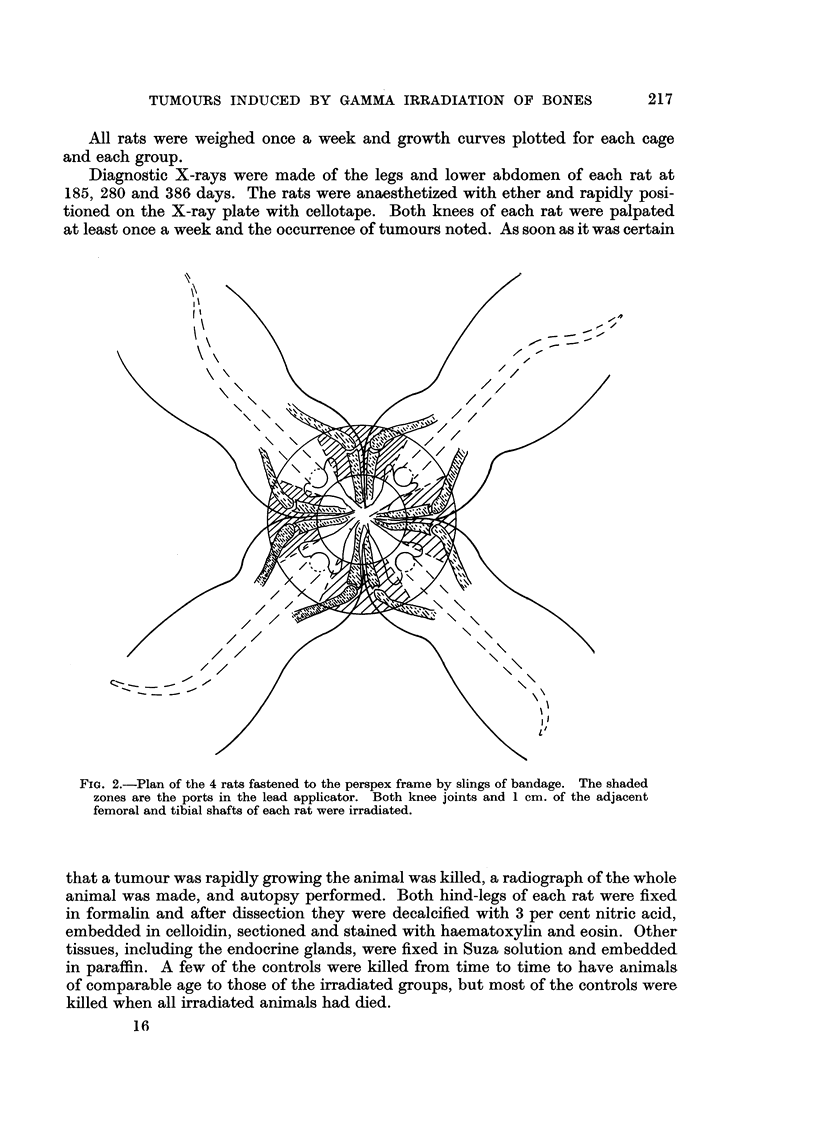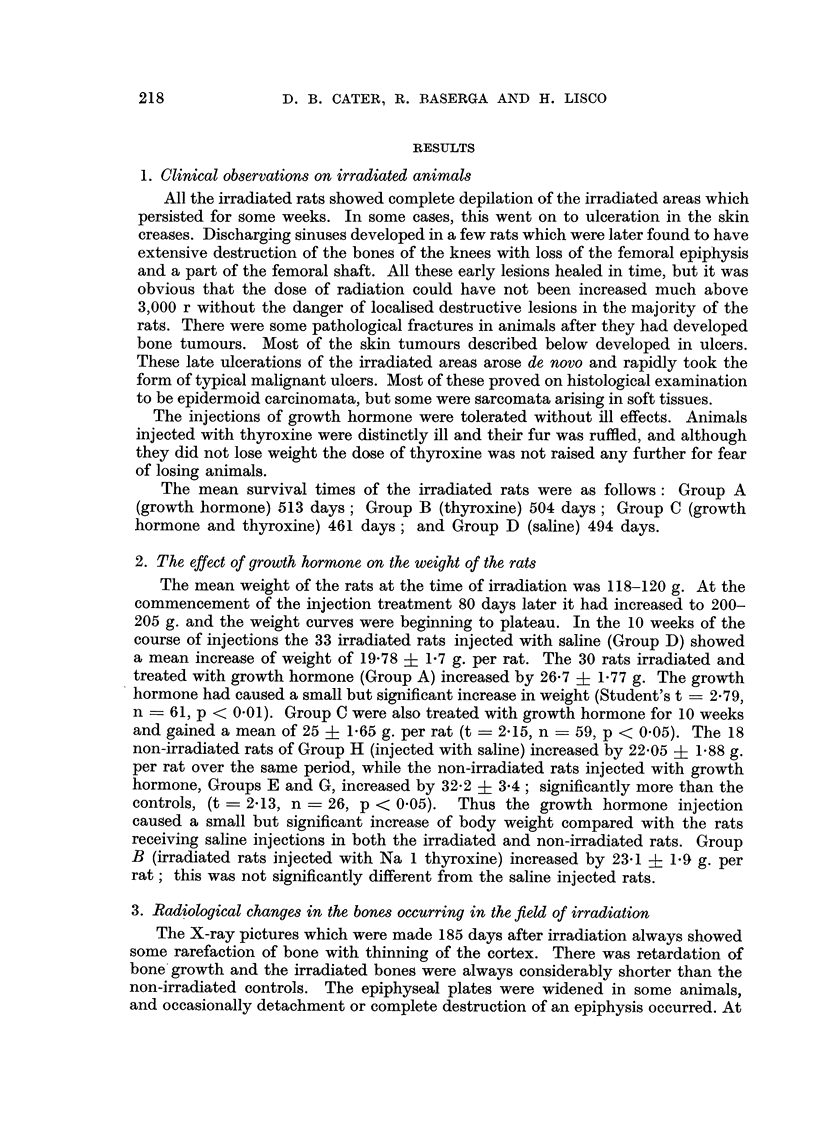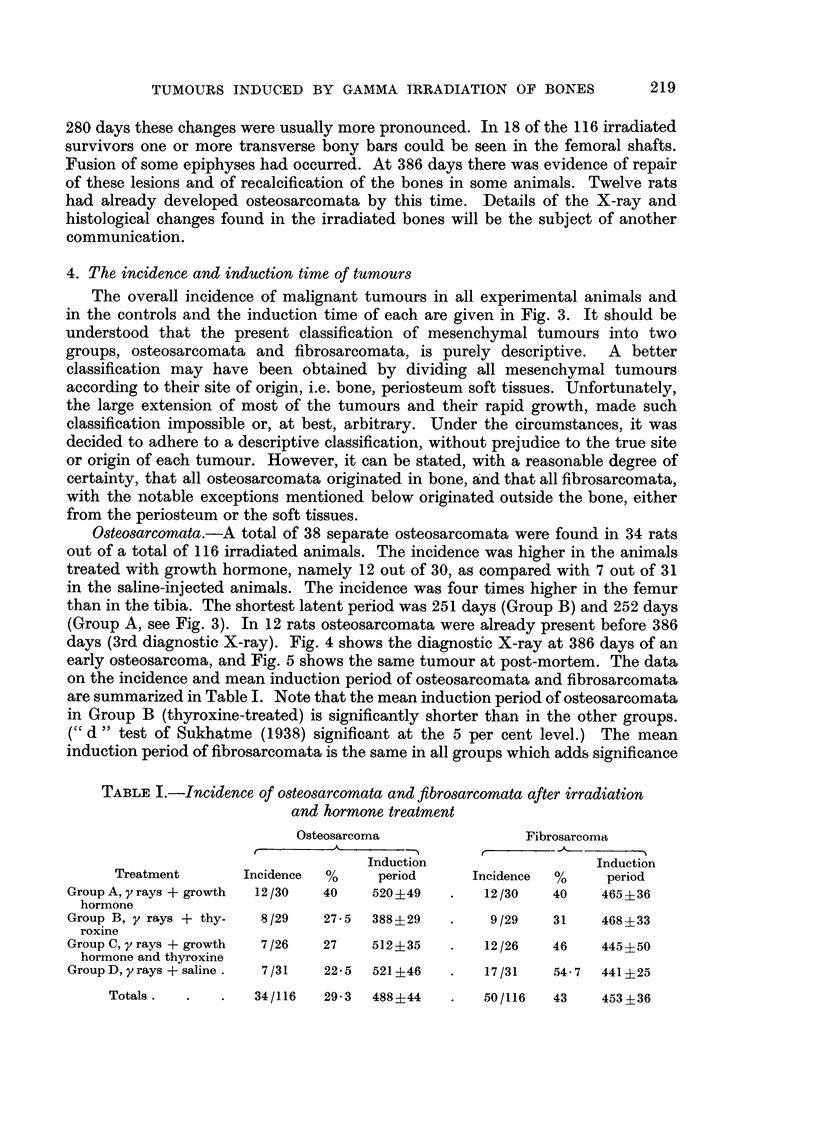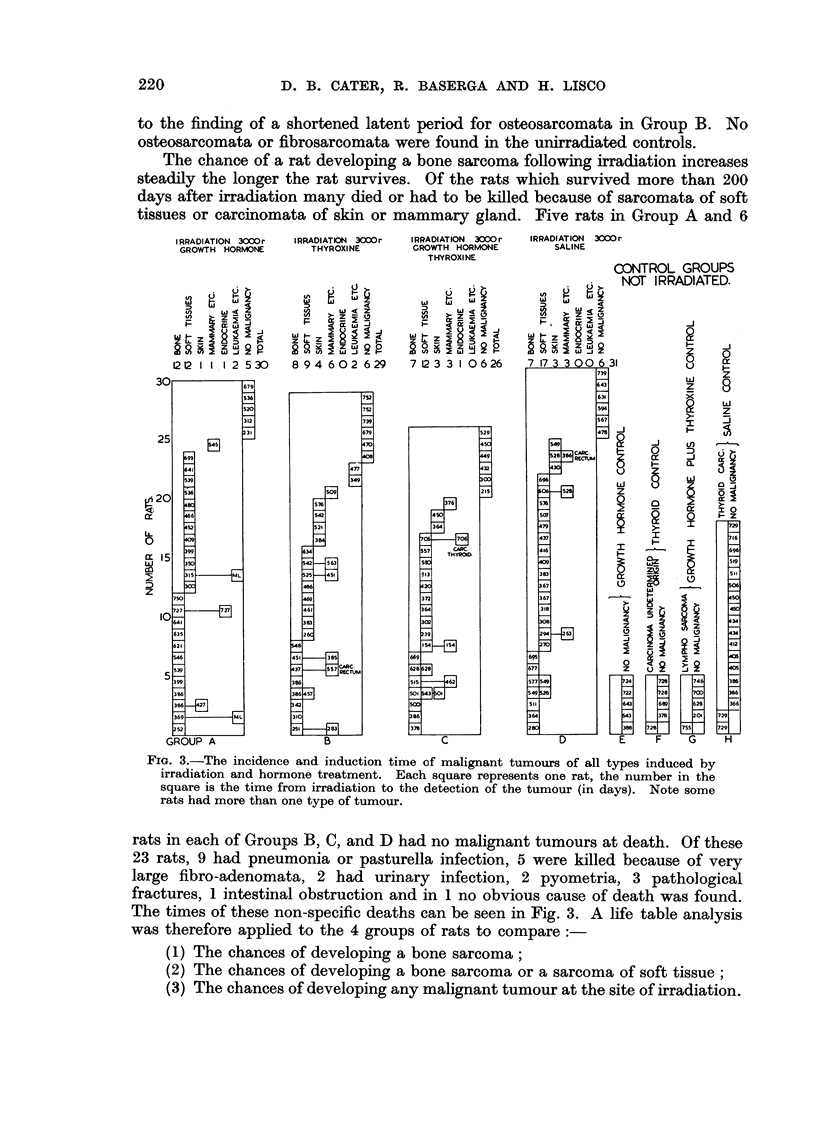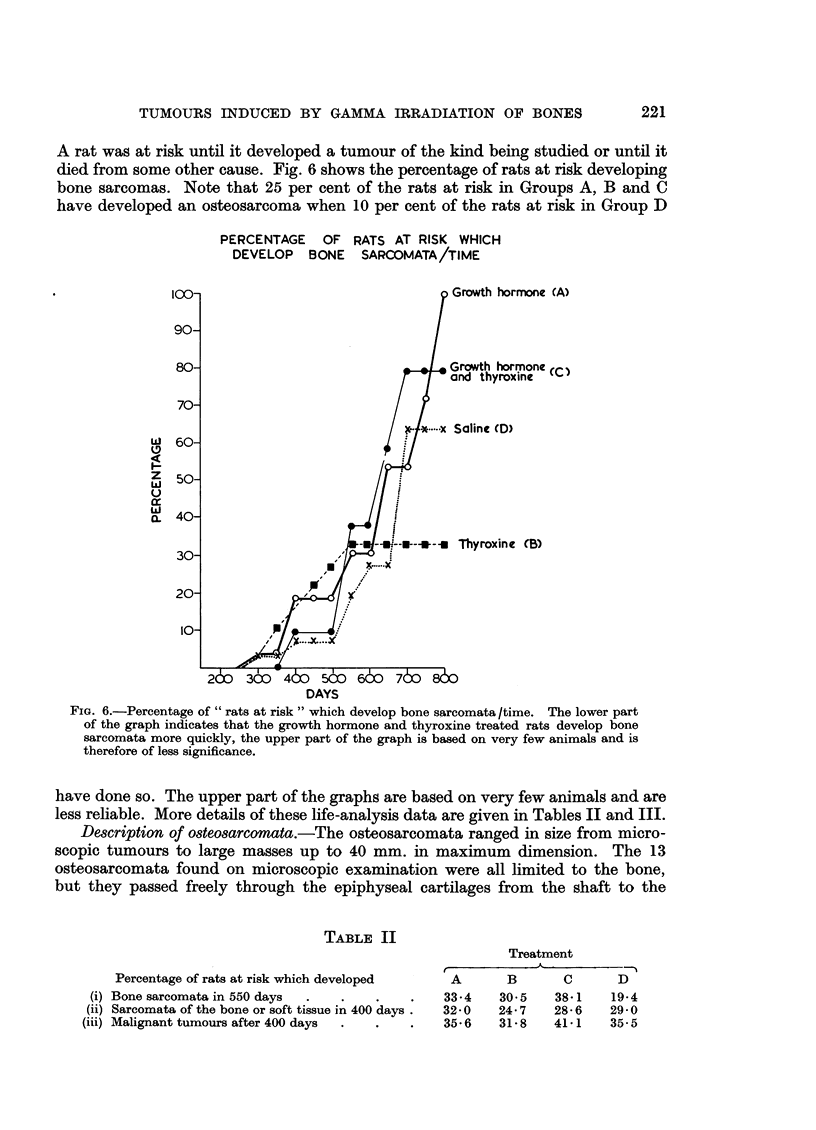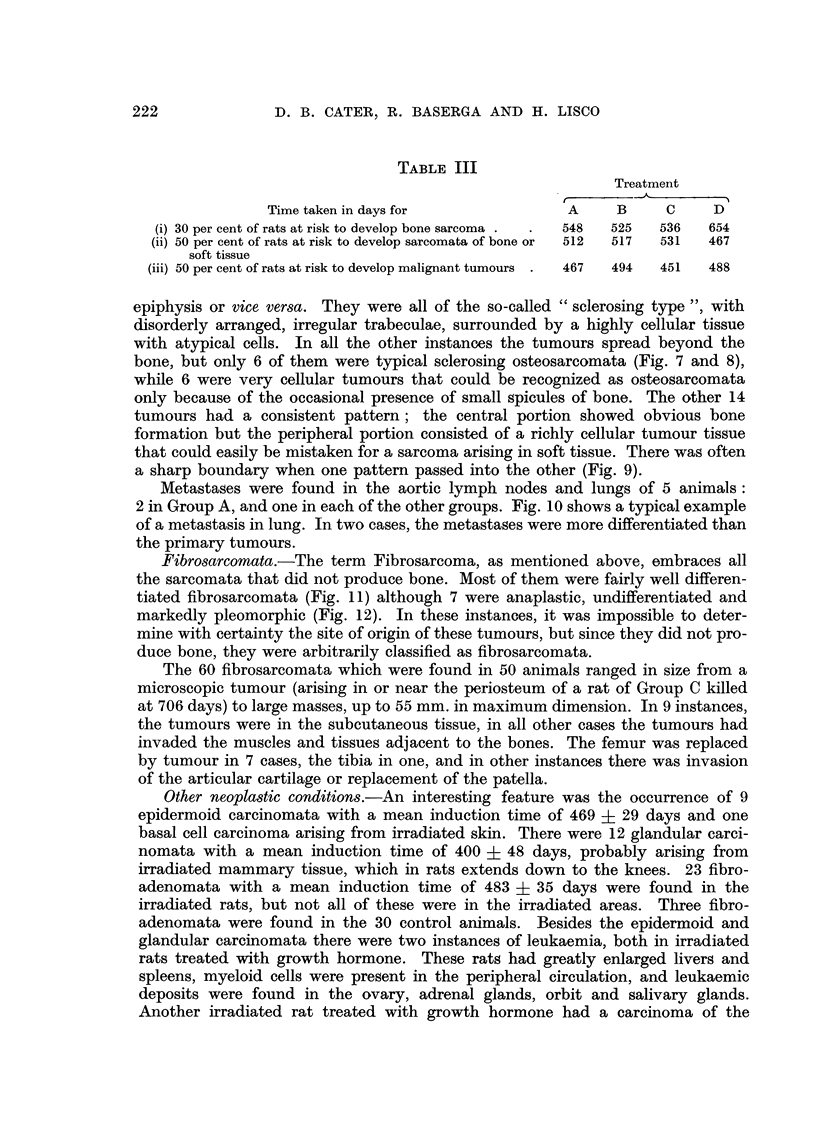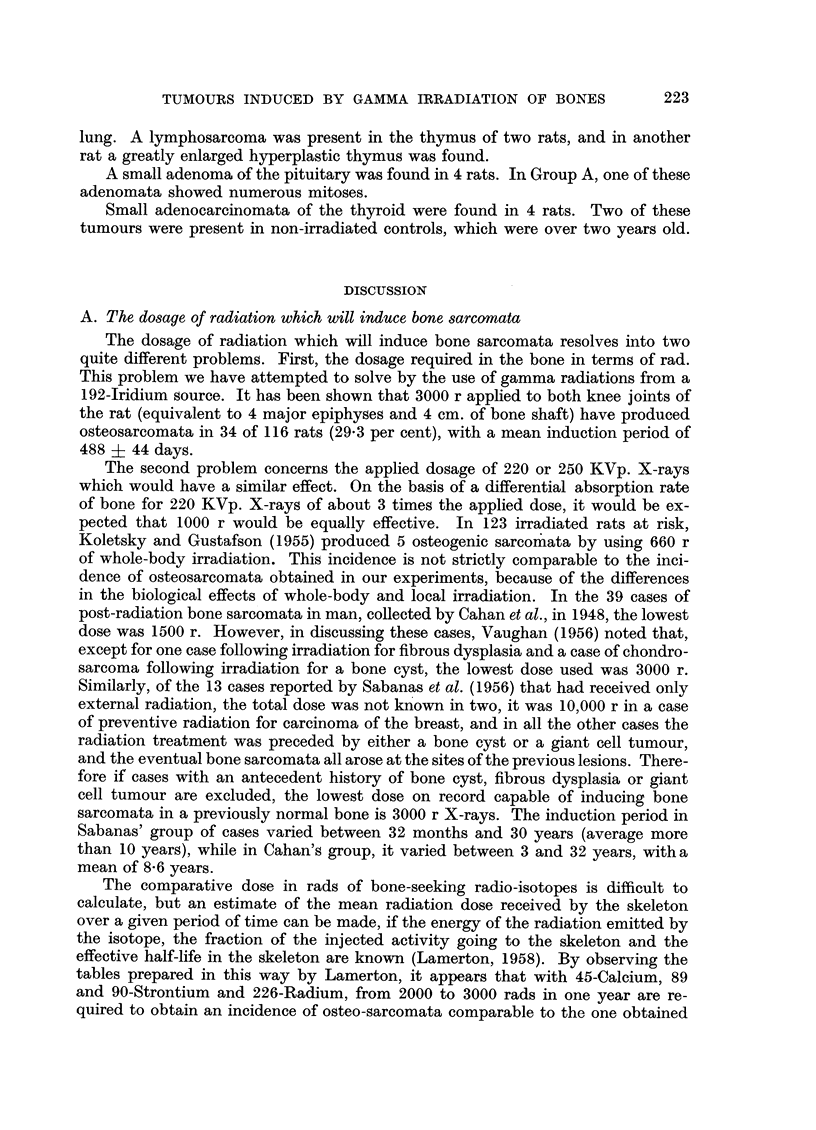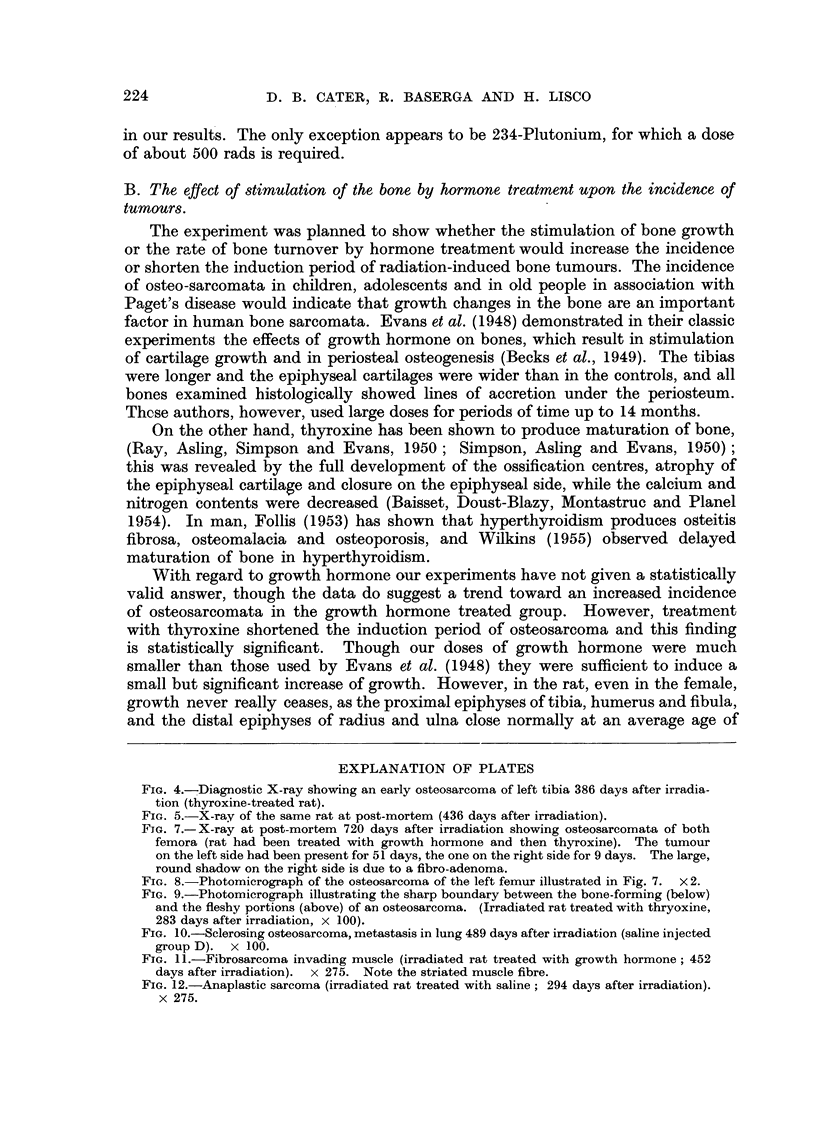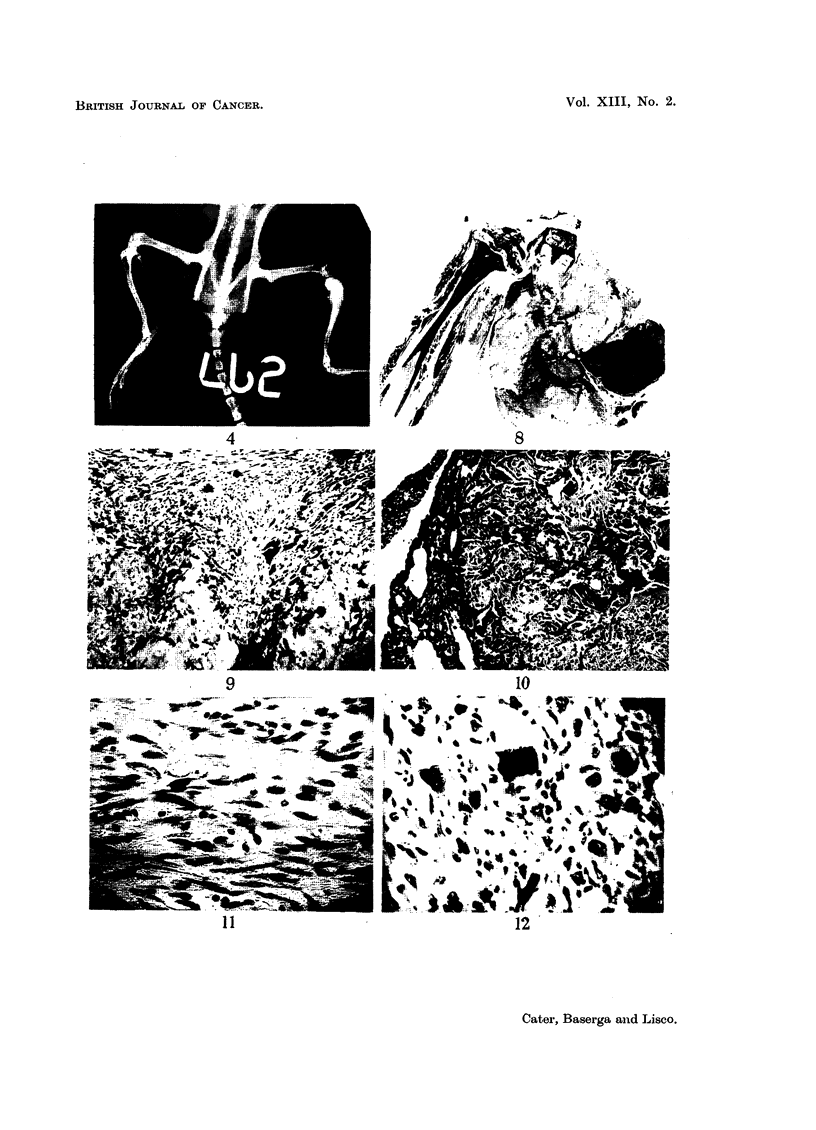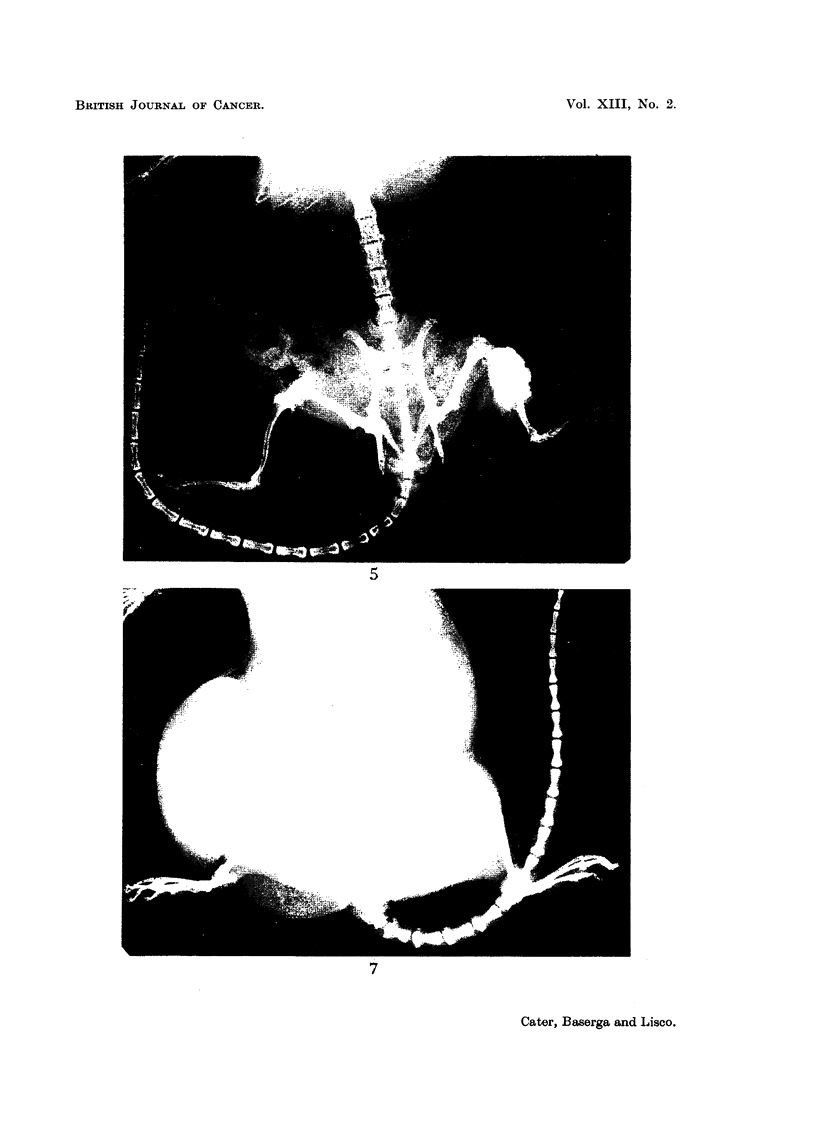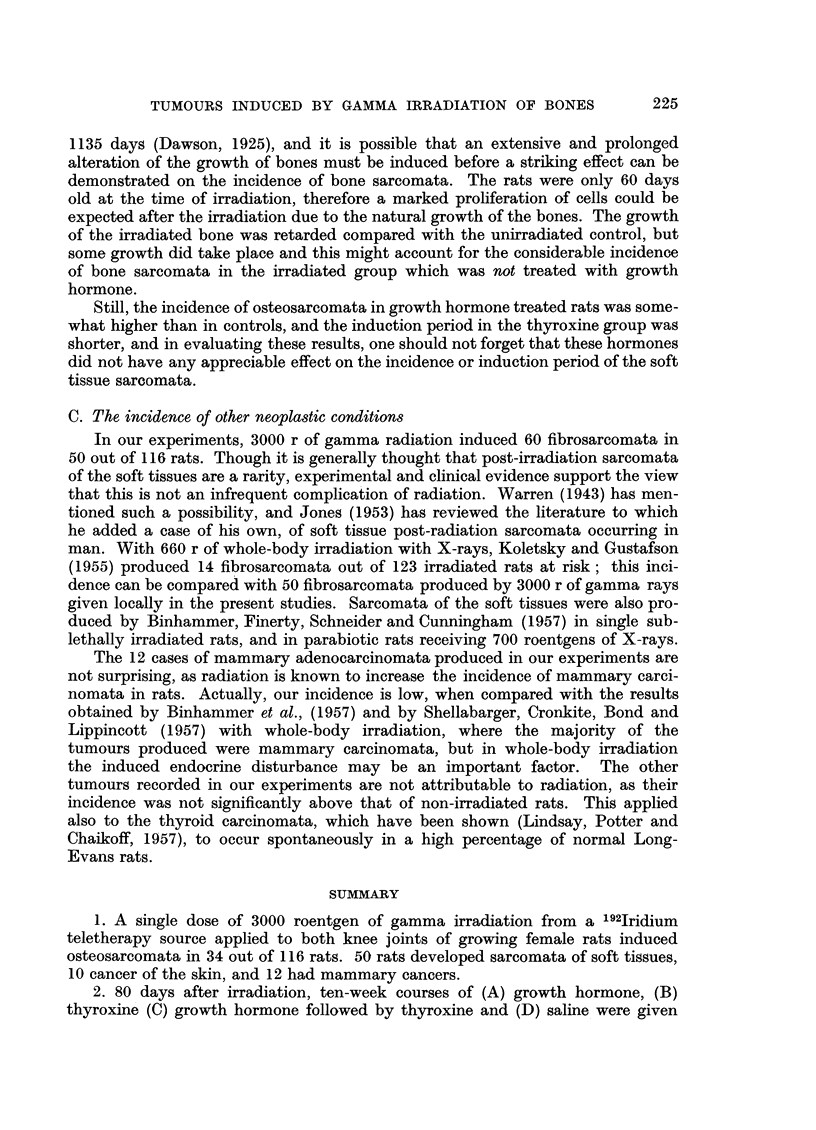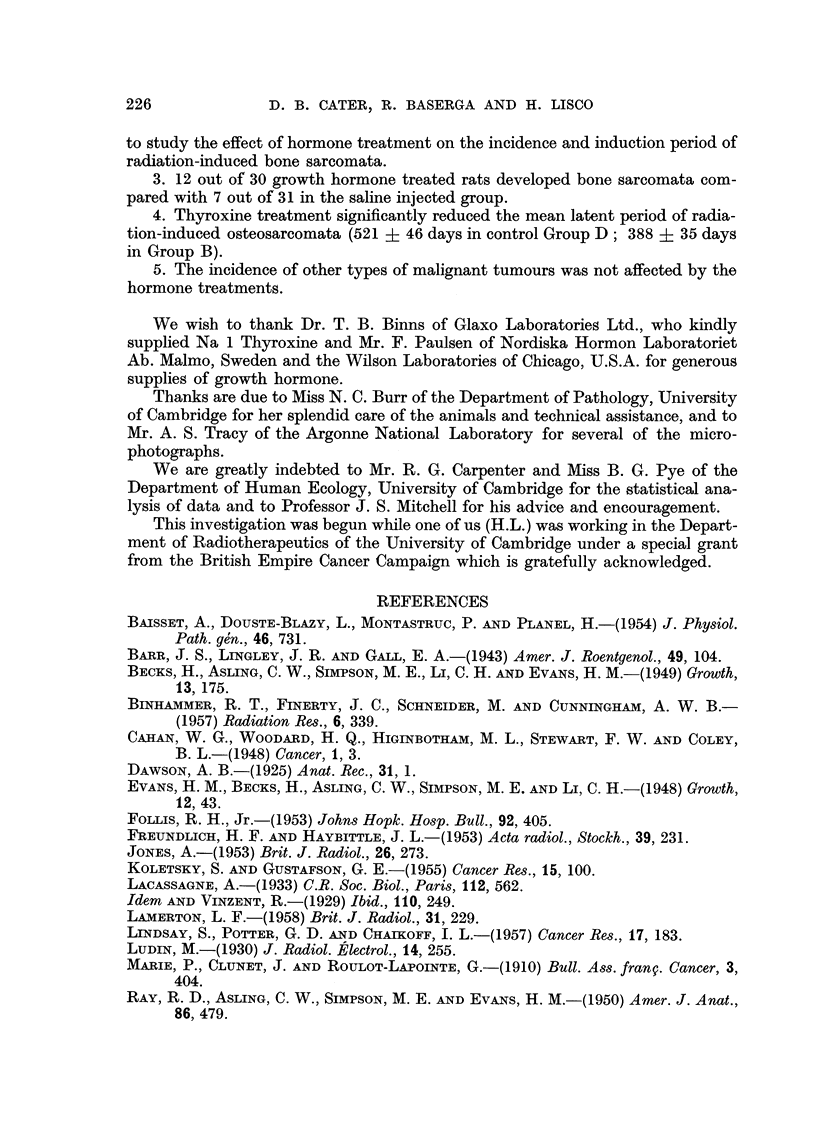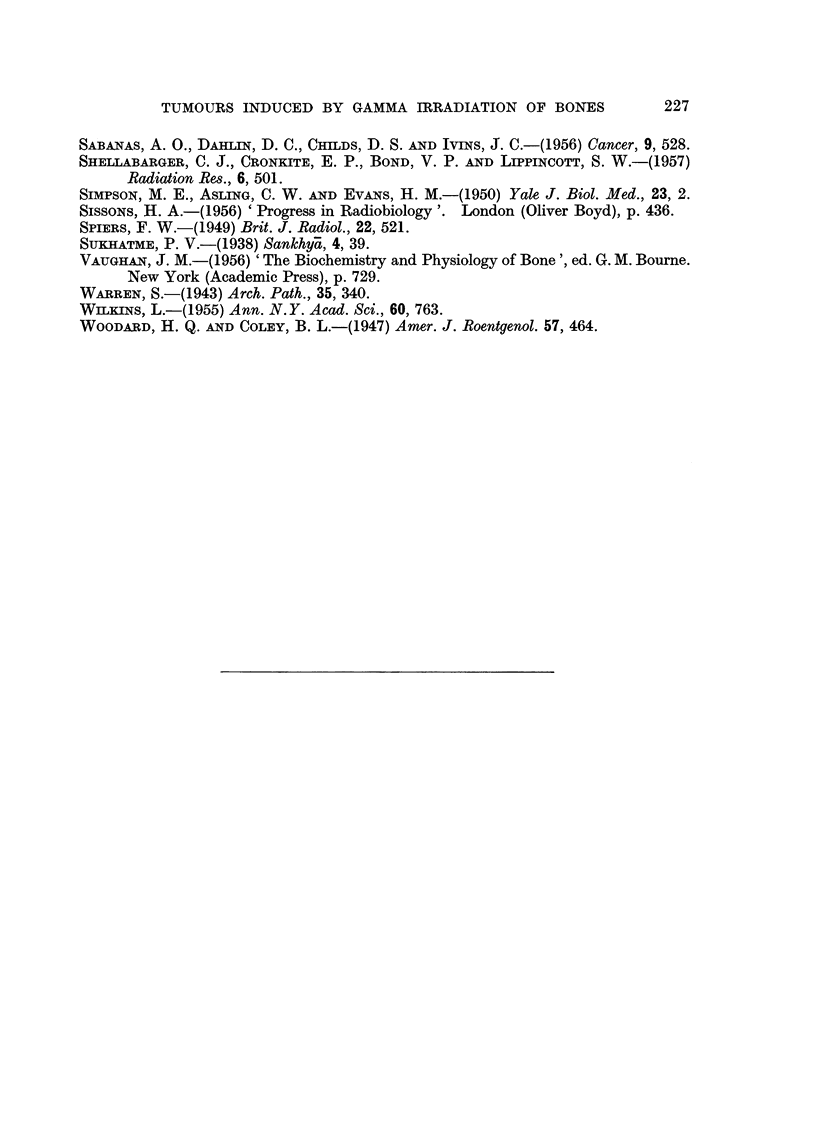# Studies on the Induction of Bone and Soft Tissue Tumours in Rats by Gamma Irradiation and the Effect of Growth Hormone and Thyroxine

**DOI:** 10.1038/bjc.1959.29

**Published:** 1959-06

**Authors:** D. B. Cater, R. Baserga, H. Lisco

## Abstract

**Images:**


### STUDIES ON THE INDUCTION OF BONE AND SOFT TISSUE TUMOURS IN RATS BY GAMMA IRRADIATION AND THE EFECT OF GROWTH HORMONE AND THYROXINE

D. B. CATER,* R. BASERGA AND H. LISCO

*From the Departments of Pathology and Radiotherapeutics, University of Cambridge, England, and the Department of Pathology, Northwestern University Medical School, Chicago, and the Argonne National Laboratory, Lemont, Illinois, U.S.A.*

Received for publication March 12, 1959

KNOWLEDGE of the dose of X- or gamma rays sufficient to induce malignant tumours in bones of humans and experimental animals is of practical and theoretical importance. Data on this subject are surprisingly limited and almost all of our information is based on not more than about 70 published cases of bone tumours in man (Sabanas, Dahlin, Childs and Ivins, 1956) and on very scanty and much less enlightening data from experimental animals. All of the bone tumours known to have been produced by external irradiation in human beings have occurred as a result of radiation therapy. The exact tissue dosages in most instances are not known, but it is apparent that all cases occurred after relatively large doses. The published figures range from 1400 to 10,000 roentgens (Cahan, Woodard, Higinbotham, Stewart and Coley, 1948 ; Jones, 1953 ; Sabanas, *et al.*, 1956).

The data on bone tumours produced by external irradiation of experimental animals are even more limited than the human data. Four malignant bone tumours have been reported after local irradiation of rats and rabbits with X-rays (Marie, Clunet and Roulot-LaPointe, 1910 ; Lacassagne and Vinzent, 1929 ; Ludin, 1930 ; and Lacassagne, 1933). The dosages again were high, but it is not possible to make reliable estimates of the tissue dosages in those experiments. In addition, five osteogenic sarcomata have been observed by Koletsky and Gustafson (1955) in a series of 123 Wistar rats exposed to 660 r of whole-body X-irradiation (220 KVp.). No other data have been found in the literature with regard to the production of bone tumours by either local or whole-body exposure to X- or gamma rays.

An estimate of the true tissue dose in the bones of either animals or human patients exposed to conventional X-rays (220 KVp.) is made difficult by the fact that the energy absorbed in bone is about 2 to 3 times greater than that in the soft tissues for the same dose of roentgens (Spiers, 1949). It is likely, therefore, that the radiation doses received by the susceptible cells in the bones of many of the cases under discussion were considerably greater, in general, than those cited in the respective papers. The prevailing uncertainties regarding the dose required to produce malignant bone tumours in experimental animals with external irradiation and the absence of any morphological studies concerning the patho-

* British Empire Cancer Campaign Research worker.

genesis of neoplasms under such conditions made it desirable to study this problem in some detail.

Accordingly we endeavoured to produce bone tumours in rats by local irradiation with a relatively large single dose of gamma rays. Gamma rays were chosen in order to avoid the aforementioned difficulties in dose estimation that are encountered with conventional X-rays. A radio-iridium source was available for the purpose. The absorption of gamma rays from iridium $^{192}$ is only about 10 per cent higher in bone than in soft tissue (as calculated from the data of Spiers, 1949). A dose of 3000 roentgen was chosen because, on the basis of clinical and experimental data (Woodard and Coley, 1947 ; Barr, Lingley and Gall, 1943 ; Sissons, 1956), it was thought that this dose would be large enough to produce severe and permanent tissue damage without at the same time causing excessive bone necrosis, fractures and other complications leading to premature death of the animals. It also seemed desirable to use a dose level somewhat comparable to the human exposures and it can be seen that 3000 r is just about twice as large as the lowest reported figure in the data on post-irradiation sarcoma in man (Cahan *et al.*, 1948). Although it would have been desirable to study the effects of at least two different dose levels in sufficient numbers of animals, this proved impractical. However, it was thought to be feasible to investigate simultaneously the subsidiary question whether hormone treatment of animals following irradiation would have a measurable effect on the induction time and yield of tumours. Bone growth and metabolism are known to be influenced by a number of hormones and since abnormalities in hormonal balance are also known to be important factors in the genesis of some tumours in other organ systems it was decided to study the influence of two hormones on the induction of tumours. The two hormones chosen for experimentation have a different and fairly specific action on skeletal tissue : (1) growth hormone stimulates the growth of cartilage (Evans, Becks, Asling, Simpson and Li, 1948) and periosteal osteogenesis (Becks, Asling, Simpson, Li and Evans, 1949) ; (2) thyroxine acts on the turnover of bone, increasing the excretion of calcium and phosphorus, and producing morphological evidence of excessive bone resorption (Follis, 1953).

### METHODS

Female Wistar rats, 60 days old and weighing between 100–130 g. were irradiated with 3000 r to both knee joints from a Radio-Iridium teletherapy unit (Freundlich and Haybittle, 1953) emitting primary gamma rays with a mean energy of 0·38 Mev. half-value layer 8·1 mm. Cu. (see Fig. 1). The dose rate varied on different occasions from 35 to 42·5 r per minute, requiring an exposure of 70 to 85 minutes. A perspex frame was made on which the anaesthetized rats could be fixed by bandage slings. Four rats were exposed simultaneously and a special lead and perspex applicator was constructed so that the 4 pairs of knee joints comprising the lower half of the femur and upper half of the tibia would be irradiated (Fig. 2). The animals were anaesthetized with either nembutal (0·1 ml./100 g. I.P.), or with sodium amytal (7 mg./100 g. I.P.). The radiation was completed in 8 sessions all performed within a period of 15 days.

One hundred and thirty six (136) irradiated rats were divided into 4 groups, A, B, C and D, and each group shared as evenly as possible the rats from the 8 radiation sessions. 34 rats from the same litters were allocated to the unirradiated control groups, E, F, G and H. Eighty days after irradiation, hormone injections were begun.

Group A of 31 rats and control Group E of 5 rats were given daily injections (except Sunday) of growth hormone subcutaneously in the flank in increasing doses : 30 $\mu$g. per rat per day for the 1st week ; 60 $\mu$g. for the 2nd week ; 120 $\mu$g. for the 3rd and the 4th week ; 240 $\mu$g. for the 5th and 6th week ; 360 $\mu$g. for the 7th week ; 480 $\mu$g. for the 8th week ; 780 $\mu$g. for the 9th week and 1 mg. for the 10th week (a total of 20·58 mg. per rat in 10 weeks). The growth hormone used was Somacton for 6 weeks, and for the last 4 weeks was a preparation made by the Wilson Laboratories, Chicago, Illinois.

FIG. 1.—Section of the radium-iridium teletherapy unit (Freundlich and Haybittle, 1953) showing the gamma-ray source, the lead applicator, and the perspex frame to which the rats were fastened.

Group B of 30 irradiated rats and Group F of 5 control animals were given daily (except Sundays) subcutaneous injections into the flank of Na 1 Thyroxine (Glaxo Laboratories Ltd.), dissolved in physiological saline containing 0·01 M sodium carbonate, 20 $\mu$g. per rat per day for 3 weeks, 30 $\mu$g. for another 3 weeks, and 45 $\mu$g. for the last 4 weeks, making a total of 1·98 mg. per rat (10 mg./kg.), over the 10 weeks.

Group C of 28 irradiated rats and Group G of 5 control rats were given a 10 weeks course of growth hormone injections identical with Groups A and E, and followed by a 10 weeks course of thyroxine injections identical with Groups B and F. Group D of 37 irradiated rats were injected daily with saline, half the group were injected for 10 weeks and half for 20 weeks. A control group H of 19 unirradiated rats were injected with saline, half for 10 weeks and the remainder for 20 weeks.

All rats were weighed once a week and growth curves plotted for each cage and each group.

Diagnostic X-rays were made of the legs and lower abdomen of each rat at 185, 280 and 386 days. The rats were anaesthetized with ether and rapidly positioned on the X-ray plate with cellotape. Both knees of each rat were palpated at least once a week and the occurrence of tumours noted. As soon as it was certain

FIG. 2.—Plan of the 4 rats fastened to the perspex frame by slings of bandage. The shaded zones are the ports in the lead applicator. Both knee joints and 1 cm. of the adjacent femoral and tibial shafts of each rat were irradiated.

that a tumour was rapidly growing the animal was killed, a radiograph of the whole animal was made, and autopsy performed. Both hind-legs of each rat were fixed in formalin and after dissection they were decalcified with 3 per cent nitric acid, embedded in celloidin, sectioned and stained with haematoxylin and eosin. Other tissues, including the endocrine glands, were fixed in Suza solution and embedded in paraffin. A few of the controls were killed from time to time to have animals of comparable age to those of the irradiated groups, but most of the controls were killed when all irradiated animals had died.

### RESULTS

### 1. *Clinical observations on irradiated animals*

All the irradiated rats showed complete depilation of the irradiated areas which persisted for some weeks. In some cases, this went on to ulceration in the skin creases. Discharging sinuses developed in a few rats which were later found to have extensive destruction of the bones of the knees with loss of the femoral epiphysis and a part of the femoral shaft. All these early lesions healed in time, but it was obvious that the dose of radiation could have not been increased much above 3,000 r without the danger of localised destructive lesions in the majority of the rats. There were some pathological fractures in animals after they had developed bone tumours. Most of the skin tumours described below developed in ulcers. These late ulcerations of the irradiated areas arose *de novo* and rapidly took the form of typical malignant ulcers. Most of these proved on histological examination to be epidermoid carcinomata, but some were sarcomata arising in soft tissues.

The injections of growth hormone were tolerated without ill effects. Animals injected with thyroxine were distinctly ill and their fur was ruffled, and although they did not lose weight the dose of thyroxine was not raised any further for fear of losing animals.

The mean survival times of the irradiated rats were as follows: Group A (growth hormone) 513 days; Group B (thyroxine) 504 days; Group C (growth hormone and thyroxine) 461 days; and Group D (saline) 494 days.

### 2. *The effect of growth hormone on the weight of the rats*

The mean weight of the rats at the time of irradiation was 118–120 g. At the commencement of the injection treatment 80 days later it had increased to 200–205 g. and the weight curves were beginning to plateau. In the 10 weeks of the course of injections the 33 irradiated rats injected with saline (Group D) showed a mean increase of weight of $19{\cdot}78 \pm 1{\cdot}7$ g. per rat. The 30 rats irradiated and treated with growth hormone (Group A) increased by $26{\cdot}7 \pm 1{\cdot}77$ g. The growth hormone had caused a small but significant increase in weight (Student's $t = 2{\cdot}79$, $n = 61$, $p < 0{\cdot}01$). Group C were also treated with growth hormone for 10 weeks and gained a mean of $25 \pm 1{\cdot}65$ g. per rat ($t = 2{\cdot}15$, $n = 59$, $p < 0{\cdot}05$). The 18 non-irradiated rats of Group H (injected with saline) increased by $22{\cdot}05 \pm 1{\cdot}88$ g. per rat over the same period, while the non-irradiated rats injected with growth hormone, Groups E and G, increased by $32{\cdot}2 \pm 3{\cdot}4$; significantly more than the controls, ($t = 2{\cdot}13$, $n = 26$, $p < 0{\cdot}05$). Thus the growth hormone injection caused a small but significant increase of body weight compared with the rats receiving saline injections in both the irradiated and non-irradiated rats. Group *B* (irradiated rats injected with Na 1 thyroxine) increased by $23{\cdot}1 \pm 1{\cdot}9$ g. per rat; this was not significantly different from the saline injected rats.

### 3. *Radiological changes in the bones occurring in the field of irradiation*

The X-ray pictures which were made 185 days after irradiation always showed some rarefaction of bone with thinning of the cortex. There was retardation of bone growth and the irradiated bones were always considerably shorter than the non-irradiated controls. The epiphyseal plates were widened in some animals, and occasionally detachment or complete destruction of an epiphysis occurred. At

280 days these changes were usually more pronounced. In 18 of the 116 irradiated survivors one or more transverse bony bars could be seen in the femoral shafts. Fusion of some epiphyses had occurred. At 386 days there was evidence of repair of these lesions and of recalcification of the bones in some animals. Twelve rats had already developed osteosarcomata by this time. Details of the X-ray and histological changes found in the irradiated bones will be the subject of another communication.

4. *The incidence and induction time of tumours*

The overall incidence of malignant tumours in all experimental animals and in the controls and the induction time of each are given in Fig. 3. It should be understood that the present classification of mesenchymal tumours into two groups, osteosarcomata and fibrosarcomata, is purely descriptive. A better classification may have been obtained by dividing all mesenchymal tumours according to their site of origin, i.e. bone, periosteum soft tissues. Unfortunately, the large extension of most of the tumours and their rapid growth, made such classification impossible or, at best, arbitrary. Under the circumstances, it was decided to adhere to a descriptive classification, without prejudice to the true site or origin of each tumour. However, it can be stated, with a reasonable degree of certainty, that all osteosarcomata originated in bone, and that all fibrosarcomata, with the notable exceptions mentioned below originated outside the bone, either from the periosteum or the soft tissues.

*Osteosarcomata.*—A total of 38 separate osteosarcomata were found in 34 rats out of a total of 116 irradiated animals. The incidence was higher in the animals treated with growth hormone, namely 12 out of 30, as compared with 7 out of 31 in the saline-injected animals. The incidence was four times higher in the femur than in the tibia. The shortest latent period was 251 days (Group B) and 252 days (Group A, see Fig. 3). In 12 rats osteosarcomata were already present before 386 days (3rd diagnostic X-ray). Fig. 4 shows the diagnostic X-ray at 386 days of an early osteosarcoma, and Fig. 5 shows the same tumour at post-mortem. The data on the incidence and mean induction period of osteosarcomata and fibrosarcomata are summarized in Table I. Note that the mean induction period of osteosarcomata in Group B (thyroxine-treated) is significantly shorter than in the other groups. (" d " test of Sukhatme (1938) significant at the 5 per cent level.) The mean induction period of fibrosarcomata is the same in all groups which adds significance

TABLE I.—*Incidence of osteosarcomata and fibrosarcomata after irradiation and hormone treatment*

| | Osteosarcoma | | | Fibrosarcoma | | |
|---|---|---|---|---|---|---|
| Treatment | Incidence | % | Induction period | Incidence | % | Induction period |
| Group A, $\gamma$ rays + growth hormone | 12/30 | 40 | $520 \pm 49$ | 12/30 | 40 | $465 \pm 36$ |
| Group B, $\gamma$ rays + thyroxine | 8/29 | 27·5 | $388 \pm 29$ | 9/29 | 31 | $468 \pm 33$ |
| Group C, $\gamma$ rays + growth hormone and thyroxine | 7/26 | 27 | $512 \pm 35$ | 12/26 | 46 | $445 \pm 50$ |
| Group D, $\gamma$ rays + saline | 7/31 | 22·5 | $521 \pm 46$ | 17/31 | 54·7 | $441 \pm 25$ |
| Totals | 34/116 | 29·3 | $488 \pm 44$ | 50/116 | 43 | $453 \pm 36$ |

to the finding of a shortened latent period for osteosarcomata in Group B. No osteosarcomata or fibrosarcomata were found in the unirradiated controls.

The chance of a rat developing a bone sarcoma following irradiation increases steadily the longer the rat survives. Of the rats which survived more than 200 days after irradiation many died or had to be killed because of sarcomata of soft tissues or carcinomata of skin or mammary gland. Five rats in Group A and 6

FIG. 3.—The incidence and induction time of malignant tumours of all types induced by irradiation and hormone treatment. Each square represents one rat, the number in the square is the time from irradiation to the detection of the tumour (in days). Note some rats had more than one type of tumour.

rats in each of Groups B, C, and D had no malignant tumours at death. Of these 23 rats, 9 had pneumonia or pasturella infection, 5 were killed because of very large fibro-adenomata, 2 had urinary infection, 2 pyometria, 3 pathological fractures, 1 intestinal obstruction and in 1 no obvious cause of death was found. The times of these non-specific deaths can be seen in Fig. 3. A life table analysis was therefore applied to the 4 groups of rats to compare :—

(1) The chances of developing a bone sarcoma ;
(2) The chances of developing a bone sarcoma or a sarcoma of soft tissue ;
(3) The chances of developing any malignant tumour at the site of irradiation.

A rat was at risk until it developed a tumour of the kind being studied or until it died from some other cause. Fig. 6 shows the percentage of rats at risk developing bone sarcomas. Note that 25 per cent of the rats at risk in Groups A, B and C have developed an osteosarcoma when 10 per cent of the rats at risk in Group D

FIG. 6.—Percentage of " rats at risk " which develop bone sarcomata/time. The lower part of the graph indicates that the growth hormone and thyroxine treated rats develop bone sarcomata more quickly, the upper part of the graph is based on very few animals and is therefore of less significance.

have done so. The upper part of the graphs are based on very few animals and are less reliable. More details of these life-analysis data are given in Tables II and III.

*Description of osteosarcomata.*—The osteosarcomata ranged in size from microscopic tumours to large masses up to 40 mm. in maximum dimension. The 13 osteosarcomata found on microscopic examination were all limited to the bone, but they passed freely through the epiphyseal cartilages from the shaft to the

TABLE II

| Percentage of rats at risk which developed | Treatment A | B | C | D |
|---|---|---|---|---|
| (i) Bone sarcomata in 550 days . . . . . | 33·4 | 30·5 | 38·1 | 19·4 |
| (ii) Sarcomata of the bone or soft tissue in 400 days . | 32·0 | 24·7 | 28·6 | 29·0 |
| (iii) Malignant tumours after 400 days . . . | 35·6 | 31·8 | 41·1 | 35·5 |

TABLE III

| Time taken in days for | Treatment A | B | C | D |
|---|---|---|---|---|
| (i) 30 per cent of rats at risk to develop bone sarcoma . . | 548 | 525 | 536 | 654 |
| (ii) 50 per cent of rats at risk to develop sarcomata of bone or soft tissue | 512 | 517 | 531 | 467 |
| (iii) 50 per cent of rats at risk to develop malignant tumours . | 467 | 494 | 451 | 488 |

epiphysis or *vice versa*. They were all of the so-called " sclerosing type ", with disorderly arranged, irregular trabeculae, surrounded by a highly cellular tissue with atypical cells. In all the other instances the tumours spread beyond the bone, but only 6 of them were typical sclerosing osteosarcomata (Fig. 7 and 8), while 6 were very cellular tumours that could be recognized as osteosarcomata only because of the occasional presence of small spicules of bone. The other 14 tumours had a consistent pattern ; the central portion showed obvious bone formation but the peripheral portion consisted of a richly cellular tumour tissue that could easily be mistaken for a sarcoma arising in soft tissue. There was often a sharp boundary when one pattern passed into the other (Fig. 9).

Metastases were found in the aortic lymph nodes and lungs of 5 animals : 2 in Group A, and one in each of the other groups. Fig. 10 shows a typical example of a metastasis in lung. In two cases, the metastases were more differentiated than the primary tumours.

*Fibrosarcomata.*—The term Fibrosarcoma, as mentioned above, embraces all the sarcomata that did not produce bone. Most of them were fairly well differentiated fibrosarcomata (Fig. 11) although 7 were anaplastic, undifferentiated and markedly pleomorphic (Fig. 12). In these instances, it was impossible to determine with certainty the site of origin of these tumours, but since they did not produce bone, they were arbitrarily classified as fibrosarcomata.

The 60 fibrosarcomata which were found in 50 animals ranged in size from a microscopic tumour (arising in or near the periosteum of a rat of Group C killed at 706 days) to large masses, up to 55 mm. in maximum dimension. In 9 instances, the tumours were in the subcutaneous tissue, in all other cases the tumours had invaded the muscles and tissues adjacent to the bones. The femur was replaced by tumour in 7 cases, the tibia in one, and in other instances there was invasion of the articular cartilage or replacement of the patella.

*Other neoplastic conditions.*—An interesting feature was the occurrence of 9 epidermoid carcinomata with a mean induction time of $469 \pm 29$ days and one basal cell carcinoma arising from irradiated skin. There were 12 glandular carcinomata with a mean induction time of $400 \pm 48$ days, probably arising from irradiated mammary tissue, which in rats extends down to the knees. 23 fibroadenomata with a mean induction time of $483 \pm 35$ days were found in the irradiated rats, but not all of these were in the irradiated areas. Three fibroadenomata were found in the 30 control animals. Besides the epidermoid and glandular carcinomata there were two instances of leukaemia, both in irradiated rats treated with growth hormone. These rats had greatly enlarged livers and spleens, myeloid cells were present in the peripheral circulation, and leukaemic deposits were found in the ovary, adrenal glands, orbit and salivary glands. Another irradiated rat treated with growth hormone had a carcinoma of the lung. A lymphosarcoma was present in the thymus of two rats, and in another rat a greatly enlarged hyperplastic thymus was found.

A small adenoma of the pituitary was found in 4 rats. In Group A, one of these adenomata showed numerous mitoses.

Small adenocarcinomata of the thyroid were found in 4 rats. Two of these tumours were present in non-irradiated controls, which were over two years old.

### DISCUSSION

### A. *The dosage of radiation which will induce bone sarcomata*

The dosage of radiation which will induce bone sarcomata resolves into two quite different problems. First, the dosage required in the bone in terms of rad. This problem we have attempted to solve by the use of gamma radiations from a 192-Iridium source. It has been shown that 3000 r applied to both knee joints of the rat (equivalent to 4 major epiphyses and 4 cm. of bone shaft) have produced osteosarcomata in 34 of 116 rats (29·3 per cent), with a mean induction period of $488 \pm 44$ days.

The second problem concerns the applied dosage of 220 or 250 KVp. X-rays which would have a similar effect. On the basis of a differential absorption rate of bone for 220 KVp. X-rays of about 3 times the applied dose, it would be expected that 1000 r would be equally effective. In 123 irradiated rats at risk, Koletsky and Gustafson (1955) produced 5 osteogenic sarcomata by using 660 r of whole-body irradiation. This incidence is not strictly comparable to the incidence of osteosarcomata obtained in our experiments, because of the differences in the biological effects of whole-body and local irradiation. In the 39 cases of post-radiation bone sarcomata in man, collected by Cahan *et al.*, in 1948, the lowest dose was 1500 r. However, in discussing these cases, Vaughan (1956) noted that, except for one case following irradiation for fibrous dysplasia and a case of chondrosarcoma following irradiation for a bone cyst, the lowest dose used was 3000 r. Similarly, of the 13 cases reported by Sabanas *et al.* (1956) that had received only external radiation, the total dose was not known in two, it was 10,000 r in a case of preventive radiation for carcinoma of the breast, and in all the other cases the radiation treatment was preceded by either a bone cyst or a giant cell tumour, and the eventual bone sarcomata all arose at the sites of the previous lesions. Therefore if cases with an antecedent history of bone cyst, fibrous dysplasia or giant cell tumour are excluded, the lowest dose on record capable of inducing bone sarcomata in a previously normal bone is 3000 r X-rays. The induction period in Sabanas' group of cases varied between 32 months and 30 years (average more than 10 years), while in Cahan's group, it varied between 3 and 32 years, with a mean of 8·6 years.

The comparative dose in rads of bone-seeking radio-isotopes is difficult to calculate, but an estimate of the mean radiation dose received by the skeleton over a given period of time can be made, if the energy of the radiation emitted by the isotope, the fraction of the injected activity going to the skeleton and the effective half-life in the skeleton are known (Lamerton, 1958). By observing the tables prepared in this way by Lamerton, it appears that with 45-Calcium, 89 and 90-Strontium and 226-Radium, from 2000 to 3000 rads in one year are required to obtain an incidence of osteo-sarcomata comparable to the one obtained in our results. The only exception appears to be 234-Plutonium, for which a dose of about 500 rads is required.

B. *The effect of stimulation of the bone by hormone treatment upon the incidence of tumours.*

The experiment was planned to show whether the stimulation of bone growth or the rate of bone turnover by hormone treatment would increase the incidence or shorten the induction period of radiation-induced bone tumours. The incidence of osteo-sarcomata in children, adolescents and in old people in association with Paget's disease would indicate that growth changes in the bone are an important factor in human bone sarcomata. Evans *et al.* (1948) demonstrated in their classic experiments the effects of growth hormone on bones, which result in stimulation of cartilage growth and in periosteal osteogenesis (Becks *et al.*, 1949). The tibias were longer and the epiphyseal cartilages were wider than in the controls, and all bones examined histologically showed lines of accretion under the periosteum. These authors, however, used large doses for periods of time up to 14 months.

On the other hand, thyroxine has been shown to produce maturation of bone, (Ray, Asling, Simpson and Evans, 1950 ; Simpson, Asling and Evans, 1950) ; this was revealed by the full development of the ossification centres, atrophy of the epiphyseal cartilage and closure on the epiphyseal side, while the calcium and nitrogen contents were decreased (Baisset, Doust-Blazy, Montastruc and Planel 1954). In man, Follis (1953) has shown that hyperthyroidism produces osteitis fibrosa, osteomalacia and osteoporosis, and Wilkins (1955) observed delayed maturation of bone in hyperthyroidism.

With regard to growth hormone our experiments have not given a statistically valid answer, though the data do suggest a trend toward an increased incidence of osteosarcomata in the growth hormone treated group. However, treatment with thyroxine shortened the induction period of osteosarcoma and this finding is statistically significant. Though our doses of growth hormone were much smaller than those used by Evans *et al.* (1948) they were sufficient to induce a small but significant increase of growth. However, in the rat, even in the female, growth never really ceases, as the proximal epiphyses of tibia, humerus and fibula, and the distal epiphyses of radius and ulna close normally at an average age of

---

EXPLANATION OF PLATES

FIG. 4.—Diagnostic X-ray showing an early osteosarcoma of left tibia 386 days after irradiation (thyroxine-treated rat).

FIG. 5.—X-ray of the same rat at post-mortem (436 days after irradiation).

FIG. 7.—X-ray at post-mortem 720 days after irradiation showing osteosarcomata of both femora (rat had been treated with growth hormone and then thyroxine). The tumour on the left side had been present for 51 days, the one on the right side for 9 days. The large, round shadow on the right side is due to a fibro-adenoma.

FIG. 8.—Photomicrograph of the osteosarcoma of the left femur illustrated in Fig. 7. ×2.

FIG. 9.—Photomicrograph illustrating the sharp boundary between the bone-forming (below) and the fleshy portions (above) of an osteosarcoma. (Irradiated rat treated with thryoxine, 283 days after irradiation, × 100).

FIG. 10.—Sclerosing osteosarcoma, metastasis in lung 489 days after irradiation (saline injected group D). × 100.

FIG. 11.—Fibrosarcoma invading muscle (irradiated rat treated with growth hormone ; 452 days after irradiation). × 275. Note the striated muscle fibre.

FIG. 12.—Anaplastic sarcoma (irradiated rat treated with saline ; 294 days after irradiation). × 275.

4

8

9

10

11

12

Cater, Baserga and Lisco.

5

7

1135 days (Dawson, 1925), and it is possible that an extensive and prolonged alteration of the growth of bones must be induced before a striking effect can be demonstrated on the incidence of bone sarcomata. The rats were only 60 days old at the time of irradiation, therefore a marked proliferation of cells could be expected after the irradiation due to the natural growth of the bones. The growth of the irradiated bone was retarded compared with the unirradiated control, but some growth did take place and this might account for the considerable incidence of bone sarcomata in the irradiated group which was *not* treated with growth hormone.

Still, the incidence of osteosarcomata in growth hormone treated rats was somewhat higher than in controls, and the induction period in the thyroxine group was shorter, and in evaluating these results, one should not forget that these hormones did not have any appreciable effect on the incidence or induction period of the soft tissue sarcomata.

C. *The incidence of other neoplastic conditions*

In our experiments, 3000 r of gamma radiation induced 60 fibrosarcomata in 50 out of 116 rats. Though it is generally thought that post-irradiation sarcomata of the soft tissues are a rarity, experimental and clinical evidence support the view that this is not an infrequent complication of radiation. Warren (1943) has mentioned such a possibility, and Jones (1953) has reviewed the literature to which he added a case of his own, of soft tissue post-radiation sarcomata occurring in man. With 660 r of whole-body irradiation with X-rays, Koletsky and Gustafson (1955) produced 14 fibrosarcomata out of 123 irradiated rats at risk ; this incidence can be compared with 50 fibrosarcomata produced by 3000 r of gamma rays given locally in the present studies. Sarcomata of the soft tissues were also produced by Binhammer, Finerty, Schneider and Cunningham (1957) in single sublethally irradiated rats, and in parabiotic rats receiving 700 roentgens of X-rays.

The 12 cases of mammary adenocarcinomata produced in our experiments are not surprising, as radiation is known to increase the incidence of mammary carcinomata in rats. Actually, our incidence is low, when compared with the results obtained by Binhammer *et al.*, (1957) and by Shellabarger, Cronkite, Bond and Lippincott (1957) with whole-body irradiation, where the majority of the tumours produced were mammary carcinomata, but in whole-body irradiation the induced endocrine disturbance may be an important factor. The other tumours recorded in our experiments are not attributable to radiation, as their incidence was not significantly above that of non-irradiated rats. This applied also to the thyroid carcinomata, which have been shown (Lindsay, Potter and Chaikoff, 1957), to occur spontaneously in a high percentage of normal Long-Evans rats.

### SUMMARY

1. A single dose of 3000 roentgen of gamma irradiation from a $^{192}$Iridium teletherapy source applied to both knee joints of growing female rats induced osteosarcomata in 34 out of 116 rats. 50 rats developed sarcomata of soft tissues, 10 cancer of the skin, and 12 had mammary cancers.

2. 80 days after irradiation, ten-week courses of (A) growth hormone, (B) thyroxine (C) growth hormone followed by thyroxine and (D) saline were given to study the effect of hormone treatment on the incidence and induction period of radiation-induced bone sarcomata.

3. 12 out of 30 growth hormone treated rats developed bone sarcomata compared with 7 out of 31 in the saline injected group.

4. Thyroxine treatment significantly reduced the mean latent period of radiation-induced osteosarcomata (521 $\pm$ 46 days in control Group D ; 388 $\pm$ 35 days in Group B).

5. The incidence of other types of malignant tumours was not affected by the hormone treatments.

We wish to thank Dr. T. B. Binns of Glaxo Laboratories Ltd., who kindly supplied Na 1 Thyroxine and Mr. F. Paulsen of Nordiska Hormon Laboratoriet Ab. Malmo, Sweden and the Wilson Laboratories of Chicago, U.S.A. for generous supplies of growth hormone.

Thanks are due to Miss N. C. Burr of the Department of Pathology, University of Cambridge for her splendid care of the animals and technical assistance, and to Mr. A. S. Tracy of the Argonne National Laboratory for several of the microphotographs.

We are greatly indebted to Mr. R. G. Carpenter and Miss B. G. Pye of the Department of Human Ecology, University of Cambridge for the statistical analysis of data and to Professor J. S. Mitchell for his advice and encouragement.

This investigation was begun while one of us (H.L.) was working in the Department of Radiotherapeutics of the University of Cambridge under a special grant from the British Empire Cancer Campaign which is gratefully acknowledged.

### REFERENCES

Baisset, A., Douste-Blazy, L., Montastruc, P. and Planel, H.—(1954) *J. Physiol. Path. gén.*, **46**, 731.

Barr, J. S., Lingley, J. R. and Gall, E. A.—(1943) *Amer. J. Roentgenol.*, **49**, 104.

Becks, H., Asling, C. W., Simpson, M. E., Li, C. H. and Evans, H. M.—(1949) *Growth*, **13**, 175.

Binhammer, R. T., Finerty, J. C., Schneider, M. and Cunningham, A. W. B.—(1957) *Radiation Res.*, **6**, 339.

Cahan, W. G., Woodard, H. Q., Higinbotham, M. L., Stewart, F. W. and Coley, B. L.—(1948) *Cancer*, **1**, 3.

Dawson, A. B.—(1925) *Anat. Rec.*, **31**, 1.

Evans, H. M., Becks, H., Asling, C. W., Simpson, M. E. and Li, C. H.—(1948) *Growth*, **12**, 43.

Follis, R. H., Jr.—(1953) *Johns Hopk. Hosp. Bull.*, **92**, 405.

Freundlich, H. F. and Haybittle, J. L.—(1953) *Acta radiol., Stockh.*, **39**, 231.

Jones, A.—(1953) *Brit. J. Radiol.*, **26**, 273.

Koletsky, S. and Gustafson, G. E.—(1955) *Cancer Res.*, **15**, 100.

Lacassagne, A.—(1933) *C.R. Soc. Biol., Paris*, **112**, 562.

*Idem* and Vinzent, R.—(1929) *Ibid.*, **110**, 249.

Lamerton, L. F.—(1958) *Brit. J. Radiol.*, **31**, 229.

Lindsay, S., Potter, G. D. and Chaikoff, I. L.—(1957) *Cancer Res.*, **17**, 183.

Ludin, M.—(1930) *J. Radiol. Électrol.*, **14**, 255.

Marie, P., Clunet, J. and Roulot-Lapointe, G.—(1910) *Bull. Ass. franç. Cancer*, **3**, 404.

Ray, R. D., Asling, C. W., Simpson, M. E. and Evans, H. M.—(1950) *Amer. J. Anat.*, **86**, 479.

SABANAS, A. O., DAHLIN, D. C., CHILDS, D. S. AND IVINS, J. C.—(1956) *Cancer*, **9**, 528.
SHELLABARGER, C. J., CRONKITE, E. P., BOND, V. P. AND LIPPINCOTT, S. W.—(1957) *Radiation Res.*, **6**, 501.
SIMPSON, M. E., ASLING, C. W. AND EVANS, H. M.—(1950) *Yale J. Biol. Med.*, **23**, 2.
SISSONS, H. A.—(1956) ' Progress in Radiobiology '. London (Oliver Boyd), p. 436.
SPIERS, F. W.—(1949) *Brit. J. Radiol.*, **22**, 521.
SUKHATME, P. V.—(1938) *Sankhyā*, **4**, 39.
VAUGHAN, J. M.—(1956) ' The Biochemistry and Physiology of Bone ', ed. G. M. Bourne. New York (Academic Press), p. 729.
WARREN, S.—(1943) *Arch. Path.*, **35**, 340.
WILKINS, L.—(1955) *Ann. N.Y. Acad. Sci.*, **60**, 763.
WOODARD, H. Q. AND COLEY, B. L.—(1947) *Amer. J. Roentgenol.* **57**, 464.